# “I am a Black man in Canada! I wish I could say differently!”: exploring the impact of direct and indirect encounters with the police and welfare system on anxiety and depression among Black youth in Toronto, Canada

**DOI:** 10.3389/fpubh.2025.1628162

**Published:** 2025-09-18

**Authors:** Eyram Adzo Agbe, Ann Kwarteng, Tricia Tetteh, Salwa Regragui, Ken Williams, Nombuso Dlamini, Godfred O. Boateng

**Affiliations:** ^1^Department of Geography and Environmental Studies, Carleton University, Ottawa, ON, Canada; ^2^Global and Environmental Health Lab, Dahdaleh Institute for Global Health Research, York University, Toronto, ON, Canada; ^3^School of Social Work, York University, Toronto, ON, Canada; ^4^Faculty of Education, York University, Toronto, ON, Canada; ^5^School of Global Health, Faculty of Health, York University, Toronto, ON, Canada

**Keywords:** mental health, Black youth, intersectionality theory, Canada, anxiety, policing, anti-Black racism, incarceration and caregiving

## Abstract

**Introduction:**

Recent studies point to differences in mental health outcomes among Black youth living in Canada, influenced by structural experiences of anti-Black racism. The influence of policing and encounters with the criminal justice and child welfare systems in Canada on Black youth mental health outcomes remains understudied, exacerbated by the minimal collection of race-based health data in the country.

**Methods:**

Based on an intersectional approach and using semi-structured interviews with twenty-four Black youth in the Greater Toronto Area, this study explores how direct and indirect encounters with the criminal justice and child welfare systems in Toronto influence anxiety and depression symptomatology among Black youth and their families.

**Results:**

We found that this population experienced significant psychosocial weathering and hypervigilance and physical insecurity, with adaptive capacity being eroded by a sense of disposability.

**Discussion:**

This study contributes new evidence to research on criminalization and racism in Canada and proposes a critical health approach to studying these issues by paying attention to the caregiving burden among Black families experiencing criminalization, as well as the influence of space and place in mitigating the health impact of police and welfare encounters.

## 1 Introduction

Globally, about 1 in 8 people experience mental health disorders. Anxiety and depression are particularly prominent, with about 301 million people living with anxiety, and 280 million people living with depression (1). Individuals who live with mental health disorders, including depression and anxiety, are at risk of experiencing a poor quality of life. Adults with depression, for instance, often have a more impaired quality of life than those with diabetes, lung disease and hypertension (2).

In Canada, the proportion of individuals suffering from generalized anxiety disorder between the ages of 15 and older has doubled between 2012 and 2022, according to Statistics Canada (3). Disaggregated by race, figures show that about 32% of people from Black communities experienced symptoms of anxiety compared to 24% of those from white communities, and 38% of Black Canadians with fair to poor mental health used mental health services compared to 50% of White Canadians (4). Canada’s Black population is young, with a median age of 29.6 years compared to 40.7 years for the total population. Children 15 years and older make up about 26% of the Black population. Recent studies show that the prevalence of depression and anxiety among Black individuals in Canada is higher than previously thought, with higher rates among those younger than 25 years of age (5, 6). A 2020 Statistics Canada study utilizing self-reported data demonstrated that among Black youth, 64% of Black girls between 12 and 17 reported excellent or very good mental health, compared to 77% of white girls in the study. Alarmingly, Black-Caribbean youth report waiting 16 months on average to access mental health care, compared to 7 months for their white counterparts (7).

The poorer mental health outcomes among Black youth in Canada are especially concerning because those between 12 and 17 years of age are three times more likely to experience moderate to severe household food insecurity than white youth in Canada, more likely to be streamed into non-traditional academic pathways than their white counterparts and are more likely to experience negative stereotypes on account of their racial identity (8). These figures point to larger socioeconomic structures that mediate race as a social determinant of Black youth’s health outcomes in Canada. As Brandt (9) elaborates, observed differences in health outcomes among racial groups demonstrate that systemic racism, rather than innate qualities, influence people’s health and well-being. Two institutions that play a major role in the manifestation of these differences in outcomes are the criminal justice and child welfare systems.

## 2 Literature review

### 2.1 Perspectives on youth policing in Canada

Across North America, the criminal justice system shapes every aspect of Black youth lives. In Canada, the Youth Criminal Justice Act came into effect in 2003 with the goal of ensuring the “rehabilitation and re-entry of a young offender into society….addressing the criticism that Canada’s youth justice system lacked a clear philosophy…” (10). Applying to youth between 12 and 17 years old, the Act stipulates that youth should not be transferred to adult court particularly before they were found guilty (which was the case prior to 2003); promotes community and extrajudicial measures instead of carceral approaches to rehabilitation; and encourages the participation of family and other stakeholders (11). Barnhorst (12) notes that while referrals are allowed instead of charges, police are likely to offer them only if youth accept unjust conditions such as taking responsibility for the offence or participating in programs that are not sufficiently tailored to them. Indeed, Marinos et al. (13) contend that the Act presents significant administrative challenges as it requires a complete shift in how police engage and make judgements about youth-involved crimes, with factors such as the seriousness of crimes, prior contact with police and youth attitudes influencing the decision to charge or divert suspected youth offenders. In a study with 70 police officers across Ontario, the seriousness of offences and prior contact with police were the two main factors mediating their decisions, with police officers being more likely to charge repeat offenders who were caught committing the same crime in the past. The Youth Criminal Justice Act has been lauded by some scholars for reductions in court interventions for offenders (especially for non-violent offences), a sound political agenda for a more equitable youth justice approach and a stronger concern for care of the public, the offender and the victim (14, 15). However, evidence on the administrative hurdles involved in implementing the Act points to structural conditions that likely shape Black youth experiences with the youth justice system.

For one, current findings about the success of the Act are not location-specific, overlooking the fact that implementation of the Act is delegated to provinces and territories across Canada, which have adopted vastly different approaches since its enactment (16). Policing cannot be separated from the socio-spatial specificities that inform its emergence, implementation and results; as geographers of policing have pointed out, law enforcement both produces the spatialities of crime with the use of technological and predictive tools (e.g., the language of crime “hot spots”), and shapes the outcomes of those who are policed with place-based valuations of social control, (dis)order and peace (17, 18).

Evidence suggests that these place-based differences in the administration of the Youth Criminal Justice Act disproportionately impact Indigenous and Black youth due to wider structural factors that influence youth involvement in crime (19). In a nationally-representative Canadian study, Fitzgerald & Carrington (20) report that minority youth, regardless of how ‘risky’ their behavior seemed, are more likely to be stopped by police than their white counterparts with the same behavior profile, pointing to the influence of race on encounters with the police that mediate experiences throughout the criminal justice system. These findings are corroborated by David & Mitchell (21) who report that Indigenous peoples are more likely to have encounters with the police as victims of a crime or as people suffering from behavioral and mental health issues. Elsewhere in a study of extrajudicial programs in the Greater Toronto Area, Samuels-Wortley (22) found that Black male youth are more likely to receive harsher punishments than males from other racial groups, and that Black youth represent 14% of the youth who were diverted or charged with cannabis possession and theft under $5,000 between 2007 and 2012. Rates of youth crime have risen in Manitoba, where indigenous youth are overrepresented due to the child welfare to prison pipeline and an overburdened justice system (19, 23), and evidence suggests that significant resources are being dedicated to reinforcing the surveillance and criminalization of racialized neighborhoods in Toronto, leading to a “spatial patterning of asymmetries” in the provision of social housing and other social reproduction infrastructure that sustains the well-being of Black youth and their families (24, 25).

### 2.2 Children’s aid and Black families in Canada

Indigenous and Black children are overrepresented in Canada’s child welfare system with resulting effects on families’ socioeconomic and interpersonal outcomes. According to the Ontario Human Rights Commission, 40.8% of the children in care at the Children’s Aid Society of Ontario were African Canadian, although they made up only 8.5% of the city’s population (26). In Ontario, where this study was conducted, Black families are more likely to be reported for mistreatment of children in their care, resulting in overrepresentation at each stage of involvement with Children’s Aid societies. Data on the influence of race-based biases in Canada’s child welfare system is still emerging, with varying theories as to the nature of discrimination that Black families face once they enter the system. King and colleagues (27), for example, note that while decision-making on Black families in Ontario did not appear to be influenced by race, there are minimal efforts to correct the “frontloading” of Black families through reporting mechanisms. Their study reflects insights by Olivo et al. (28) and Antwi-Boasiako et al. (29), who argue that factors such as oversurveillance of Black families through application of the Child Protection Standards, structural inequality (which manifests as poor socioeconomic outcomes among Black families) and lack of awareness on cultural differences in parenting influence Black families’ higher risk of involvement in the child welfare system.

While further research is necessary to establish the extent to which racial disparities influence decisions made regarding families in the welfare system, recent qualitative studies conducted among case workers and impacted parents and youth paint a worrying picture about the treatment of racialized families. In a study of 27 Black youth who were placed in out-of-home care away from their families, Edwards and colleagues (30) highlighted how transracial placements with families that lacked cultural sensitivity, poor communication from case workers about the cause and duration of youth’s placement, and dismissal of youth’s needs (such as appropriate hair care and familiar meals) influenced feelings of exclusion and exhaustion. Participants in this study noted that extended separation from loved ones and an apparent resistance to placements with Black families weakened youths’ ability to cope with emotional and material challenges such as feelings of loneliness and lack of housing. Elsewhere, youth reported feeling disposable and emotionally exhausted as they were constantly relocated with their belongings in trash bags (31). Frequent movement and short-term stays at foster care homes, characterized as placement instability, contributes to long-term behavioral challenges among youth independent of the effects of the conditions around their entry into the welfare system, although said instability often goes hand-in-hand with adverse childhood experiences like exposure to domestic violence and incarceration of a family member (32, 33). Research delineating pre-existing adverse childhood effects from placement instability is currently rare in Canada, but this remains a critical public health concern for youth as data from the United States demonstrates that placement stability contributes to improved emotional well-being, reduces the risk of youth engaging in recurring criminal behavior and possibly mitigates the risk of post-traumatic stress disorder among affected youth (34).

Black parents’ interactions with the welfare system are no less frustrating, as other studies reveal that in some cases, parents who attempt to advocate for their children are labelled as aggressive or uncooperative, exacerbating the stereotype of the angry Black man/woman (35). It is worth noting that Black families often lack access to information about the welfare system; like many of the young people in other qualitative studies, they feel that they have no institutional support to navigate what became an overwhelming, longstanding engagement with numerous stakeholders (36). In effect, many Black youth in Ontario’s welfare system arguably exist in a constant survival mode, resorting to extreme strategies to cope with new living arrangements or to get much needed institutional attention (such as acting out or running away from home), representing an additional psychosocial burden that impedes youth and their families’ capacity to heal and thrive as a collective (31).

### 2.3 Underscoring the mental health impact of encounters with police and the welfare system

As Simon (37) elaborates, racial discrimination is naturalized through everyday discourses that filter from above *and* below, even as dominant power seeks to undermine the pervasiveness of contemporary forms of racial discrimination. This bears significance for the construction of the Canadian state and its reproduction of young Black lives. Canada is widely constructed as a beacon of social welfare with the Medicare system and other social supports, a reputation belied by the double bind of racial capitalism and settler colonialism that undergird the nation’s foundational systems (38). This is no less the case for healthcare and is reflected in the fact that Black youth typically access mental healthcare when engaging with the justice system or have cases severe enough for intervention (39). The analysis of the criminal and welfare systems demonstrates that stakeholders and policies are implicated in constructing Black youth and their families as risky, suspicious and disposable. This “white racial frame,” which prioritizes the worldview and decision-making process of white and dominant stakeholders, naturalizes otherwise contentious narratives that translate into troubling outcomes for various marginalized groups (40). It is also important to contextualize these material impacts of discourses to the increasing role of the police as health stakeholders, a consequence of the transition to community-based care that is rooted in the withdrawal of the welfare state in the 1970s in Canada (41, 42). Displacing health policy into the arena of “public safety” has proven fatal for Black youth, and it is worth investigating how these realities – amidst efforts to expand for-profit care and long wait times in healthcare facilities in Ontario (43) – influence lived experiences of policing.

So far, existing data on policing and the welfare system in Canada points to the growing relevance of the concept of weathering in understanding the mental health effects of encounters and their resulting procedural issues. According to Arline Geronimus, observed racial health disparities cannot simply be explained by factors like genetics and exercise, as the stress of living in impoverished conditions as a racialized person wears down on the body’s biological systems, causing numerous health problems down the line as a result of premature ageing (44). This weathering hypothesis is attentive to how intersecting axes of oppression produce noticeable neurological symptomatology that lead to poor mental health outcomes for marginalized groups, particularly among lower-income Black women (45, 46). Here, stress and one’s coping strategies influence the weathering effect; in a study on the impact of stress and heart disease among African-American women in Chicago, Warren-Findlow argues that by coping with the stress of disease and poor access to healthcare with narratives of strength and resilience, many participants’ symptoms worsened as they minimized the extent of their illness to meet ongoing familial and community obligations (47). The weathering hypothesis then recognizes that interpersonal and relational tools, in addition to radical institutional change, are essential to resolving the accelerated aging and negative health consequences exacted by long-term exposure to race-based health inequalities (48). Long wait times, fear of institutionalization and geographical disparities in access to youth mental health care across Ontario exacerbate risk factors for mental illness in the province. However, studies also point to misconceptions about community perceptions of people living with mental illnesses, religion and practitioner relationships as barriers to healthy coping mechanisms among racialized Canadian youth (49). These institutional and interpersonal issues, then, are worth analyzing in the context of a mental healthcare regime where many racialized youths and their families only access care at the point of crisis and go on to survive both their diagnoses and treatment at the hands of service providers (50).

Youth mental healthcare in Canada needs a dynamic shift. Disruptions to brain development during adolescence can drastically impact youth vulnerability to the risk of mental health disorders, particularly since environmental risk factors such as socioeconomic discrimination can influence the onset of mental health disorders (51). Thus, this study contributes to a growing area of research in Canada that explores how the justice system influences Black youth health outcomes. Specifically, using a qualitative research approach, we explore how direct and indirect experiences with the criminal justice and welfare systems in Toronto influence Black youth’s mental health outcomes and shape their coping strategies. This study seeks to understand, in the youth’s voices, the social structures informing their encounters with police and the welfare system, the experience of said encounters and the mental health impacts resulting from them.

## 3 Theoretical framework: intersectionality theory

This study draws on intersectionality theory to analyze the mental health outcomes of Black youth who have had experiences with these systems, as well as their coping strategies. Intersectionality theory recognizes that lived experiences are shaped by their multiple identities, which are in turn influenced by structural inequities such as health racism, over policing and inadequate mental healthcare systems. An intersectional analysis allows researchers to analyze how individuals experience multiple forms of discrimination; as Crenshaw (52) posits, this prevents an atomization of people’s experiences based on distinct parts of their identities, allowing for a more nuanced understanding of how the interlocking nature of their identities compounds discrimination. Critically, intersectionality theory recognizes how people within various sub-groups have differential access to power, which then shapes their positionality to other members of their sub-group and the collective experiences of social capital and discrimination that they experience.

Intersectionality theory is relevant to this study because many of our participants, who come from immigrant, racialized backgrounds embody identities that uniquely influence their day-to-day experiences. For example, 1 in 5 racialized families in Canada live in poverty, compared to 1 in 20 non-racialized families in a phenomenon known as the racialization of poverty. This trend is feminized as well, with racialized women earning 32% less than non-racialized counterparts (53). This raises questions about the well-being of low-income, female-headed racialized households, for instance, especially those impacted by incarceration or involvement with the child welfare system. Furthermore, Black youth in Toronto are routinely constructed as dangerous, at-risk individuals whose communities pose an existential risk to the safety of the city. Here, racialized youth in Malvern, Jane and Finch and other “priority neighborhoods” are marked as stigmatized individuals whose challenges are products of specific areas that must be contained away from the rest of the city. These narratives overlook how structural conditions both create and reinforce “problem areas” and underserved youth (54). Thus, in this study, an intersectional analysis attends to how “certain ideas and practices surface repeatedly across multiple systems of oppression and serve as focal points or privileged social locations for these intersecting systems” (55). An intersectional lens into how direct and indirect encounters influence mental health outcomes among youth in the Canadian context is central to challenging the misconception that these encounters are value neutral, undertaken per due process and free of the systemic racism that many in dominant society believe is more routine in the United States. This analysis specifically calls for continuous engagement with the endurance of settler colonialism and anti-Black racism in the establishment and workings of Canada’s various institutions, as we argue that the conditions that result in poor mental health outcomes for Black youth and their families are neither new nor insurmountable. Rather, they constitute legacies of intertwined, interlocking social struggle within Indigenous and Black communities, manifesting in the use of space, access to social services and a sense of belonging.

## 4 Materials and methods

This study was conducted in the Greater Toronto Area, a large metropolitan area divided into five regions (Durham Region, Halton Region, Peel Region, Toronto Region and York Region). Demographic information demonstrates that there are about 400,000 self-identified Black people in the GTA, representing 7% of the area’s total population. Black residents of the GTA are also increasingly located outside the City of Toronto and in more suburban parts of the Greater Toronto Area and Toronto hosts about 150,630 Black youth out of a total of 240,000 as reported in 2021 (56). Crucially, Black people are significantly overrepresented in crime rates in Toronto, with Black men being 3.7 times more likely to be charged for crimes (with charge rates between three to seven times higher than that of white men) (57). Wortley & Jung (57) also note that the significant overrepresentation of Black people in these statistics points to high rates of police surveillance and likely use of more violent policing strategies in racialized communities. The Ontario Human Rights Commission also reports that Black people were involved in over 28% of use of force cases and 61.5% of deadly encounters with the police between based on data from 2000–2006 and 2013–2017, despite constituting 8.8% of Toronto’s population in 2016 (58). Elsewhere, Black youth in a study of lived experiences of Blackness in the Greater Toronto Area reported being stopped by police more than being helped by them, with about 54% of participants stating that police in the GTA did not treat Black people fairly (59). These data point to tenuous experiences of policing for Black Toronto youth, contesting its youth-friendly image (60). Youth in Toronto also report fear of criminalization and hospitalization, alienation from mental healthcare service providers and a complicated healthcare system as barriers to accessing mental healthcare, demonstrating intersections with the criminal justice system (61). While the Anti-Racism Act of 2017 mandates the collection of race-based data by public sector organizations in Ontario, data collection and standardization is in its infancy across many institutions (62, 63). This calls for more place-specific research that engages Black youth perspectives in a practice of counter-storytelling, toward disputing mainstream narratives about their lived experiences, and elevating (inter)personal and communal practices that support their health and well-being amidst experiences of stigmatization by the police and welfare system (64).

### 4.1 Ethics statement

The studies involving human participants were reviewed and approved by the Research Ethics Board at York University (Certificate #: e2024-026). Written informed consent to participate in this study was provided by the participant’s legal guardian/next of kin. Written informed consent was also obtained from the participants and the legal guardians of minors for the publication of any potentially identifiable images or data included in this article.

### 4.2 Participants and data collection

A purposeful sampling approach was used to recruit participants across the Greater Toronto Area (GTA). Purposive sampling is useful in studies where rich, in-depth information is necessary for answering the research question at hand (65). With this approach, sampling requires “a series of strategic choices about with whom, where and how one does one’s research,” beginning with concrete decisions about what the researcher wants to achieve and the kind of knowledge they intend to generate (66).

Twenty-four Black youth and their families between the ages of 17 and 39 years of age with direct and indirect experience were recruited for this study. Of this figure, 21 participants were youth between the ages of 17 and 30 years of age, while 3 participants were parents and caregivers between 34 and 39 years of age. Direct encounters in this study are conceptualized as “face-to-face communication with an officer, more than a passing greeting” (67). This includes police stops (traffic stops, being stopped on the street or other location), raids, being questioned by case workers at school and being admitted into a foster home. Indirect or vicarious encounters include those who have seen others experience these scenarios, without involvement themselves, or heard about others’ experiences. For the purposes of this study, we asked participants to describe their most memorable experiences with the criminal justice and child welfare systems regardless of the period they occurred in.

Gashanti and Ghana Union, the community partners in this project, reached out to community members, partners, and social workers to recruit youth and families that fit our criteria. Given the exploratory nature of the study, emergent sampling techniques were also employed. Specifically, we used community educational campaigns and information sessions to recruit more young Black men with direct experience with the police and/or welfare system, given initial difficulty with recruiting from this demographic, and the adverse psychological impact of encounters with the police on them (68). This proved particularly productive, as participants discussed some of their reservations with participating in a study of this nature, highlighting the language used in participant recruitment and distrust for research on mental health and policing. Snowball sampling was also instrumental in recruiting participants, as those who had participated and felt at ease referred friends and family to the researchers (69).

Once participants agreed to participate in the study, they completed relevant ethics protocol documents. They then filled an online survey comprising two scales measuring depression and anxiety symptomatology (Impact of Event Scale and Generalized Anxiety Disorder-7 Scale) and proceeded to the qualitative portion of the study. Semi-structured interviews lasting between 30 min and 2 h were conducted by staff from Gashanti, Ghana Union and the York University research team at participants’ location of choice (online or in-person). While separate interview guides were prepared for those with direct and indirect experience, and family members of those with direct experience, participants’ responses touched on questions across both guides depending on the nature of their experiences. The semi-structured interviews included 12 demographic questions, 2 major questions on experiences as racialized people in Canada and life histories (particularly related to immigration stories), as well as 15 questions on encounters with the police and welfare and community dynamics. The semi-structured interview guide included prompts to encourage both the interviewer and participant to explore lines of inquiry that were not immediately obvious with the initial questions. Interviews with youth and families were conducted separately to ensure that participants felt comfortable sharing their experiences with the research team. Participants were free to skip any questions they did not feel comfortable answering. Interviews were audio-recorded with participants’ consent or recorded with field notes where they preferred.

Participants interviewed with members of the research team who had closer cultural proximity to them, meaning that most participants from the Somali community in this study spoke to the Gashanti team, and participants from Ghanaian backgrounds spoke to the Ghana Union team and some research team members from York University. Although the insider/outsider binary in qualitative research has been challenged (70, 71), it was critical to include insiders in this study to facilitate a closer sense of rapport and trust with the research process. Our institutional partners in this project also occupied “the space between” in some cases, as their experiences did not neatly line up with those of some participants (72, pg.100). Where participants were more comfortable speaking to someone outside their cultural context, they were referred to the York team for the interview. All interviews with the York team were conducted online using the Zoom platform, or via phone call.

### 4.3 Data analysis

After data collection, interviews were transcribed orthographically by two research assistants and the research coordinator, using a shared template. This naturalized transcription approach captured participants’ body language, breaks in speech, slang and original speech. This approach preserves the socio-cultural signals present in participants’ speech and adds to the richness of the data collected (72). Transcripts were reviewed for accuracy and formatting by the research coordinator.

Data were then analyzed on MAXQDA24 using thematic analysis, with the first author reading the transcripts for familiarity (especially for those interviews conducted by community partners) and generating preliminary codes on life in Canada, experiences of policing, mental health and coping strategies (73). After review, these codes were then stratified into themes on individual, interpersonal, communal and institutional factors shaping encounters with the police and welfare system, mental health outcomes and adaptive strategies. It is worth noting here that the data analysis process did not occur in isolation with the author and the transcription software alone; field notes, ongoing conversations with community partners and preliminary notes following orthographic transcription all shaped the themes explored in this article. In this paper, quotes are reported with pseudonyms to protect the identity of participants.

### 4.4 Ensuring rigor

Rigor in qualitative research is critical in determining the quality of one’s research findings; in the absence of specific criteria to determine whether qualitative findings are trustworthy, the data lose meaning and hold little applicability for policy and community application (74, 75). Given that this research is an exploratory study meant to influence subsequent phases of a larger project, the research team incorporated Lincoln and Guba’s (76) criteria for ensuring rigor in qualitative enquiries throughout the research process.

#### 4.4.1 Credibility

During the interview process, the research team continually engaged with social support workers, community organizers and youth leaders who were interested in the research but not actively involved in the day-to-day of data collection. This allowed the research team to understand how professionals external to the project perceived its objectives, questions and recruitment material, informing reflexive changes to the recruitment strategy and the language used in the interview questions. The research teams also incorporated negative case analysis by actively seeking out insights that differed from emerging observations, specifically by recruiting more individuals who had direct experiences with the criminal justice system and those with direct experiences with the child welfare system.

#### 4.4.2 Dependability and confirmability

To ensure a coherent audit trail, regular meetings on project logistics were held between community partners and the York University team, all documented and disseminated with meeting minutes. Revisions to the interview guides were tracked and documented using word processing software, and a transcription guide was developed by the research team to ensure that all transcripts were uniform for coding and analysis. During the transcription process, interviews were transcribed by three people and reviewed collaboratively to ensure conformity with the audio recordings and transcription guides. One-on-one meetings were held with community partners after interviews and information sessions to debrief, discuss emerging findings and ascertain what type of changes were necessary to advance the data collection process.

## 5 Results

### 5.1 Weathering due to administrative and social burden of managing encounters with the police

Many participants in this study expressed physical and mental weathering, or compounding levels of stress that negatively influenced their well-being because of the amount of administrative and social work involved in managing the outcomes of an encounter with the police and/or welfare system. These effects were most notable among Black female adult participants who supported youth who had direct experiences with these systems or community members, although this weathering effect was also noticeable among Black boys and young men who had direct encounters with the police. Here, we noticed that both the encounter and proceedings after the fact contributed to the physical and psychosocial weathering effects that participants experienced.

Farha’s procedural experiences with the police after she witnessed the shooting of a 15-year-old by another Black youth and reported the perpetrator of a crime in her neighborhood points to why Black youth across this study were vulnerable to the weathering effect of police encounters even where police were seemingly out to protect their health and well-being. This participant felt conflicted about reporting the crime because the perpetrator’s older brother was involved with gangs, prompting fears of retaliation against her younger brother. Her attempts to explain why she felt uneasy about helping the police went unheard, as she recalls that:

I realized that I was talking to them about my fears and safety of my brother but all they were responding was about their case. So they weren’t understanding my issue. So, that’s when I realized there’s no point further explaining just understand their issue…they didn’t care. I was more…I remember even telling them like, honestly, I missed a 20% assignment because it was due the same day I saw [the shooting]. I was like, I was too distraught to even like, you know, like…I was talking to them about schooling and all they could respond with…yeah but we need to get this case airtight.

Although she tried to minimize her feelings about the police treatment (which included relentless emails sent to her university email address) her account of how her emotions unfolded point to how this experience only contributed to wearing down her adaptive capacity. She described her emotions as a glass bottle with beads that broke when she finally reckoned with the compounding effects of witnessing the shooting and dealing with her father’s untimely death. That her treatment at the hand of the officers contributed to tipping her ‘bottle’ highlights an erosion of the duty of care they have toward citizens, contributing to a sense of mistrust and reluctance to participate in the ‘justice’ process, especially since the prioritization of the case seemed to constitute an injustice against her psychosocial well-being.

Solace, a justice worker of Somali descent had two brothers with direct encounters with the criminal justice system, with one being diverted to a mental health institution where they eventually passed away. Due to the high cost of proper representation and the reluctance of about five lawyers to take one brother’s case, her family resorted to pursuing a monetary settlement for her mother as a form of closure. Speaking about the long-term impact of this trauma in her current role as an advocate for Somali families whose young people encounter the police and mental health systems, she highlighted her challenges with adopting healthy coping strategies with her family:

And sometimes you know my kids can be like, “Mom you seem like you’re tense. Are you okay?” Or sometimes my children will be like: “You’re on the phone all the time, why are you not giving us attention?” And that’s when I realize at the end of the day, I also need to understand when I’m a mom, I’m a mom. And I need to park my work hat at work. So, you know, it’s not more so of a safety issue, it is more so a timing issue where I need to be a bit more vigilant and be more respectful of how I’m adequately giving my time to my family.

Here, it is evident that the longstanding effect of witnessing one’s relatives being arrested and charged, and later taking on the responsibility of managing the administrative and psychosocial impact of these encounters, bleeds into Solace’s family life. Her advocacy exceeds temporal and emotional boundaries, and this quote implies that the difficulty of ‘knowing’ when to be a mum constitutes an additional mental boundary with likely consequences for her children.

Pat expressed how her brothers’ entanglement with the criminal justice system beginning when she was 13 years old had an ongoing effect on her family’s physical and mental health. The police barging in at night to arrest her brothers “gave [everyone] shock,” and the ongoing criminal proceedings had a particularly harrowing cascading effect on her family due to her parents’ inability to navigate the criminal justice system, maintain their health and parent their other children. An adopted cousin attempted suicide due to the lack of parental care and loss of his older brothers as role models, as “he’s always sad and angry, just like me.” Her parents’ mental and physical health points to an intergenerational weathering effect of youth encounters with the criminal justice system:

My mother’s health is failing. You know, she's worried, and we're constantly worried about her. We're telling her… there's nothing you can do, right? Don’t worry about it. At the moment, I have one brother that’s in jail right. So, she’s worried. What if he gets beaten up inside? What if he's not getting fed? What if the crime is not him that did it? You know, she’s forever worried. My father has just shut down. He’s not as talkative as he used to be. He’s not as happy as he used to be. It's just like this whole thing got to him. He’s just tired.

Her mother’s concerns about her brother’s circumstances extend to his presence in jail and his treatment there, pointing to how enduring the systemic and discursive strategies that shape encounters with the police and experiences in the court system are – especially for a family that also faced significant hurdles in acquiring legal and mental health support. The weathering effect of these encounters was especially pronounced for this family because their parents’ capacity to provide for their children was worn down by the abrasiveness of their encounters with the police. As a result, there was a devolution of care work and social support for this participant, who was not only “forever worried herself,” but also noted that her brothers’ absence meant she and her sister had to take up the cultural responsibilities that they could not assume as men in the family. This created financial pressures in their households, further intensifying their feelings of regret and resignation toward their brothers. Here, it is important to note that while participants were able to identify how structural factors influenced their loved ones’ rationale for engaging in criminal activity, direct involvement with the police themselves and the day-to-day heaviness of cutting through the legal system led to conflicting feelings about being put in these situations by their loved ones.

### 5.2 Hypervigilance, physical and social insecurity

Another predominant theme across the data was that Black youth who had both direct and indirect experiences with the police resulted in feelings of hypervigilance because these encounters had radically transformed their perceptions of where and how they would be profiled, arrested or criminalized. Sal, a 20-year-old university student of East African descent, grew up in an overpoliced neighborhood in North York where they were frequently stopped and questioned by police, noted that they automatically assumed they had done something wrong when they saw police around, because the police only ever came for them:

Interviewer: You mentioned whenever you see the cops, you automatically think it’s coming for you or your friends?

Sal: I have to, I have to, yeah.

Interviewer: Have to?

Sal: Yeah, because in my head, I’ve never seen a police officer come for anything else except for an investigation, to ask us questions, accuse us of something, right. I’ve never seen them come to actually help us. That’s always in my head. It’s always they’re against us, right. And I don't know how to explain it but that’s how…

It was imperative for them, for instance, to ask whether the police were there for their family or friends if they noticed their presence near their home, as a matter of self-preservation. This mental orientation did not emerge out of thin air, as he described how he had been harassed by Toronto police on a summer day while they were with their friends downtown:

And then they came up to me saying, “Open your bag”. Like, as if I have a weapon on me or something because I fit some description. You know? But it was annoying, and I was thinking, “Like, why did I open my bag? Like why? They didn’t have the right to search me?” I didn’t have to open it. But then I was scared, if I didn’t open it, what would happen, right?

This quote demonstrates that the process of making these assessments about police and adjusting behavior to protect oneself is an incredibly taxing one. Wondering why he had been hounded by the police manifested in questioning himself and his choices, with the quote implying a sense of regret at failing to stand up for oneself in the face of police injustice. Anda linked this experience to the role that police play in the city’s spatial and political landscape, often comparing the roles of the police to security guards, firemen and other ‘first responder’ occupations. She worked with a youth community group and frequently travelled to Toronto’s downtown core, noting that she was often hesitant to help homeless people and people in crisis because of the police. Specifically, she would simply walk away from the scene in order not to be visible to police, an experience that white people may not necessarily have:

I don't think they are consciously aware, or that some are made to be aware that the system is made for them. So, when they're in contact with police officers, they're not thinking about the other things I'm thinking of, like, what if they lash out at me. Because why would a public servant lash out at a civilian because, like, they are the civilian, I [police] am not the civilian. I’m not that person that they're meant to serve and protect. I feel like they [White people] understand they're the blueprint on that stuff. So, they [White people] feel more comfortable putting them [police] in their place.

That this participant felt that white people were not consciously aware of this dynamic with the police also meant they did not have to spend time thinking about how to negotiate their identities in interactions with law enforcement. Pointing out how police did not see them as civilians and someone worth protecting demonstrates a fundamentally broken social contract between police and Black communities in Canada, succinctly linked to the history of policing in North America as an institution meant to protect the interests of slave owners. The comparison with other first responder/enforcement groups, then, serves to distinguish them as “people who help” and do not “interact with people and their rights and their well-being, like police officers have the ability to.”

Not all youth felt uneasy or hypervigilant around police, with some noting that they felt that they provided a sense of security and would only make them anxious if they had had previous encounters with them. Others were more ambivalent about the presence of police in their communities, noting that they would either mind their business or seek out alternative forms of resolution if they were ever in a situation that would ordinarily require a police response:

Um…it like with the area that I'm in…if I'm being harmed, I'd probably like reach out, like, call the police. But also if I saw someone else like, I mean I would but…that wouldn’t be the first initial thought that comes to my mind. The first thought would just be try and mind your business as much as possible.

This participant, who was 16 and of Jamaican descent who was robbed and subsequently not taken seriously by the police, was especially taken the qualitative decline in his mental well-being following the incident. While she categorized her experience with the police as an indirect one, she had also been stopped by police while she was on a walk with two male cousins because the officers were trying to pin a crime reported in the community on the two boys. Thus, her inclination to mind her business may have been influenced by the anxiety she expressed at the treatment of Black boys in her community, and how this had pushed her to become an advocate in her social settings.

Across these participants’ responses, navigating the reality of racial discrimination in one’s day-to-day life involves seemingly minute calculations about avoiding the possibility of encounters with the police or welfare system in the first place. While these strategies may seem inconsequential on a daily basis, research on microaggressions in the United States demonstrates that ongoing exposure to stressors related to racial discrimination is associated with lower rates of physical activity, increased risk of cardiovascular illness, and poor behavioral coping strategies (77).

### 5.3 Disposability and the limits of ‘community’

For youth who had been incarcerated or had longstanding interactions with the criminal justice system, there was a significant sense of sadness and anger as they had been abandoned or disposed of by people in their community, whether they were friends or members of their cultural community. Chris, who was in court over a firearms charge, noted that it took about one month for friends and some family members to cut off contact with him after they realized the possible severity of his sentence. To him, it appeared that they had rationalized that he was going away for a long time and was no longer useful to them as a friend or loved one. He wistfully recalled how status as persona non grata made him lonely and isolated by choice, as he was not sure who would stick around if they knew about his charges. His experience communicates the ambivalence of ‘community’ for Black youth with direct encounters with the police and welfare systems, as it quickly becomes unclear who is for/against them, and what community means in the first place – particularly for Black male youth like this participant who had lost parental figures to police violence and struggled with feelings of loss, social inclusion and anxiety.

Disposability strained the limits of community in supporting the adaptive capacity of youth and their families after encounters with the police and welfare system. Community was a salient theme in relation to how people’s social status shifted after encounters because from some accounts, the influence of religious leaders, friends, family and supportive law enforcement officials significantly eased participants’ sadness, stress and long-term weathering connected to the encounter. Kingsley, who was enrolled in a restorative justice program after getting into a violent fight, recalled how community support positively affected him. His counsellor and the church he volunteered at wrote letters of support as he progressed through his program, a gesture that reminded him of the importance of the values they were instilling in him:

Interviewer: Um…so, these encounters with these people in positions of power, what would you say has been the impact on your sense of self? Has it been a good one?

Kingsley: I feel like it kind of reminded me of who I was. I looked back at stuff they taught me at my middle school (the Afrocentric one). It really showed me that what they’re saying was the truth and stuff like that. It helped me because when I looked back, I used some of the stuff.

The absence of this support was especially deleterious for those participants who had ongoing encounters in the court and welfare systems because the amount of information required to navigate both systems for the best outcomes and the parameters of accessing more rehabilitative options depended on significant involvement from one’s community. Some participants who expressed moderate to significant difficulty with managing the mental health impact of their encounters with the police noted uncertainty with the idea of community because they had grown up watching adults in their community – and now, their own generation – experiencing and acting on feelings of paranoia, insecurity and overprotection. For instance, Palou noted that growing up, “everything was kinda sectioned off” based on parents’ relationships with each other, a sentiment that was only compounded after friends who lived in social housing were dispersed across the city by the Children’s Aid Society after a police raid on their apartment. It is worth noting that this sense of insecurity and overprotection on the part of parents was influenced by the socio-spatial manifestation of policing, as he expressed growing increasingly vigilant of wearing headphones, being outside past with a group of friends and enjoying public spaces due to increasing attention from the police. Moreover, he and other participants recognized that their parents navigated significant material and psychosocial gaps in their responsibilities, heightening their own feelings of exclusion. While participants cited community BBQs, grassroots organizations, sporting groups and religious events as examples of their communities being “less separated,” there was a sense of uneasiness about the durability of these relationships, especially since the conditions that complicate feelings of trust amongst neighbors are structural and extend outside communities. Liliana, a Jamaican Canadian youth who entered out-of-home foster care in middle school due to parental abuse, noted that:

So like, when there were issues, there was no calling the police. I think it’s only when it gets to a serious high point, that’s when they may turn to it [police]. But in terms of like their [community] discipline and parenting tactics, it is very harsh. There is no grace. I don't think they have any understanding of what grace is. And especially like in the areas that we grew up in and what we really needed.

Also, they didn’t have the tools needed to know what we needed. So, I feel like that’s a big factor…The thing also is that people don’t change, or they just do the same thing. Like there’s teenage moms who have their kids and then their kids become teenage parents, things kind of just keep happening.

Liliana’s commentary on how immigrant parents in her community did not know how to even anticipate their children’s needs reflects the literature on the challenges of immigrant parents raising children in new countries while carrying values, expectations and practices from their previous experiences, both in their home countries and in the host country (78). It is interesting that in this case, parents’ inability to identify what their children needed and extend grace to them emerged from their own feelings of exclusion because of their familial status as single or teenage parents. Living through intergenerational trauma exacted within community weakened their ability to navigate racial trauma from dominant society, with second generation children being the collateral damage of poorer coping strategies among their parents. Aside the fact that this alienated them from formal and informal support networks, this finding also highlights the limits of resilience among immigrant, racialized communities in this study. Physical and psychological resilience are crucial to helping immigrants assimilate into new cultures, as experiences with institutional trauma and distance from established community are significant stressors among this demographic. That parents in this quote seemed incapable of dealing with various dimensions of said acculturation stress raises the importance of recognizing resilience as a multi-faceted process that involves social, ecological and biological processes and has consequences for the mental well-being of second generation immigrants (79). Thus, it is crucial to interrogate easy narratives of adaptation and resilience that celebrant immigrants’ capacity to cope with the burden of moving, raising families in a new culture and establishing themselves anew, as this quote demonstrates that there are massive limits to this demographic’s capacity to thrive especially when their symbolic psychosocial strengths are taken for granted in public health discourses.

## 6 Discussion

In this article, we argue that encounters with the criminal justice and welfare systems in the Greater Toronto produce a weathering effect on youth and their families. This effect is noticeable in the resulting physiological and psychological symptoms that mothers, fathers and sisters experience in the wake of encounters, as well as how they compound over time to strain their families’ capacities to navigate the systems at hand. Figures suggest that 12.4% Black Canadians live in poverty (compared to 8.1% of the general population) with the poverty rate being double that of white people in the third generation or more. Additionally, 33% of Black children between 0 to 14, and over 26% of Black youth between 15 and 24 lived in low-income housing in 2016. In the same year, Black youth aged 23 to 27 were less likely than other groups of Canadian youth in the same age range to have a postsecondary certification, diploma or degree (8). Toronto recorded a child poverty rate of 24.5% among Black people, higher than the non-racialized average of 9.1% and the racialized average of 17.8%.

These figures demonstrate that many of the Black youth and families in our study and across the city are under significant socioeconomic stress that produces and influences their life outcomes as racialized people. For families that have encounters with the justice and welfare systems, these stressors are exacerbated by the bail system (where the accused risk being held in remand for long and sometimes falsely plead guilty to gain their freedom) and the cost of legal representation (80). This theme also highlights the psychosocial impact of care provided by ‘captive maternals’, who “provide the reproductive and productive labor to stabilize culture and wealth.” As Joy James elaborates, they are tied to the state even as they care for others in response/resistance to it, highlighted by the time theft that marks the experiences of relatives of incarcerated people. This time theft (i.e., the empirical loss of time to do life’s work of picking up children, cooking and doing the laundry from the time spent advocating and pursuing legal justice) weathers those who provide this vital care as they lose the opportunity to recover from the trauma and stress of not only navigating the police and court systems, but also bearing the impact of systemic discrimination in their everyday lives. As the cases of the two sisters of incarcerated Somali young men show, the “reproductive and productive labor [of their families]…” act as a subsidy for the myriad health and social services that remain unavailable to families navigating the criminal justice system (81).

The analysis also demonstrates the disposability of Black youth in the criminal justice and welfare systems, which informs their feelings of anxiety, hypervigilance and social exclusion. Cacho (82) argues that among marginalized people, criminality and criminalization are often conflated, meaning that some groups are treated as if they have already committed future crimes and may as well have done so without witnesses, because they are seen as being fundamentally criminal. As a result, these groups are rendered “illegible for personhood” (82), a condition implied throughout participants’ hypervigilance about being immediately suspected of a crime by police because they are either not regarded as children and young citizens/residents of Toronto worth protection by the police and welfare system. Moreover, these sentiments seem to manifest within community settings, further eroding participants’ capacity to bounce back from involvement in the criminal justice system. It is worth noting that the displacement of young people who have been through the criminal justice system from communal spaces also manifests spatially, as Black youth are aware of the ways that they are circumscribed within certain places in the city and within their neighborhoods. That this treatment extends to parents navigating the criminal justice and child welfare systems presents a challenge to Ontario’s and Canada’s justice and public health systems. Specifically, the growing recognition of the role that systemic racism plays in influencing outcomes in these systems is the first step toward resolving the inhumanity that racialized Canadians experience. It is crucial for these institutions to integrate culturally sensitive approaches that firstly recognize the humanity, vulnerability and strengths of racialized communities in Canada. Echoing recent statements from members of the Canadian Association of Child and Adolescent Psychiatry, effecting these changes primarily means establishing a cogent research agenda for studying the links between experiences of racism in Canada’s institutions and mental health outcomes among racialized families and their children (83). However, we caution that efforts to better understand these links should be undertaken with care, in recognition of the significant emotional, social and psychological costs associated with the care work and survival that families navigating these systems experience daily. While an increased diversity among social workers, police officers and foster families is a welcome recommendation, our analysis demonstrates that mere diversity without a deep cultural understanding and capacity to wield one’s institutional power in support of better health outcomes is problematic. Resolving the hypervigilance, weathering effects and fractured community relationships among racialized communities in the criminal justice and child welfare systems begins with an institutional ethic of care that values meaningful representation and ensures that those who encounter these systems are not further burdened by what should be restorative, reconstructive processes.

## 7 Limitations

Our study had few participants who had experience with children’s aid/child welfare system in Ontario. Among the few who did have this kind of experience, participants were reluctant to talk about their experiences and mostly preferred to talk about their experiences with police or law enforcement. Recruiting families and individuals with experience in the child welfare system requires a culturally sensitive approach as involvement with this arm of social services is seen as especially embarrassing based on feedback from community members. Thus, while we could not include these perspectives in this study, this feedback suggests that there is an ongoing mental health impact on Black families and children that have been involved in the child welfare system that have potential generational health effects.

## 8 Conclusion

The findings from this exploratory study point to the relevance of two sites of further inquiry. Firstly, the relationship between caregiving and criminalization among Black families remains undertheorized. Evidence in this study that social reproduction among Black families is compounded by the crisis of capital as well as the weathering effect of balancing the administrative hurdles of the criminal justice process, family and one’s personal psychosocial well-being. Thus, further studies could assess how “caregiving at the margins” influences the socioeconomic well-being of Black youth and their families. We contend that there are opportunities to study the caregiving burden on youth, as evidence demonstrates that young people take on advocacy roles based on their experiences being criminalized or watching friends and family encounter go through the criminal justice system. Research in this domain could involve the development of caregiver burden scales that recognize the intersectional, interlocking impact of administrative, health-related and institutional factors on Black families’ capacity to care for their loved ones involved in the welfare or criminal justice systems.

Another line of inquiry that we identify from this exploratory study is a critical assessment of the role that space and place play in influencing encounters with the criminal justice system and mitigating mental health effects among Black youth. Our study demonstrates that Black youth are presumed to be “out of place” in key arenas of the city, with resulting impact on their mental and social well-being. While the neighborhood effects literature demonstrates that poor social infrastructure in a given community negatively impacts one’s life outcomes, there is a need for more critical perspectives connecting the construction of space and the structural factors influencing said neighborhood effects. We argue that an intersectional approach to studying the relationship between race, spatiality and the criminalization of Black youth involves studying the intersection and disjuncture between urban planning, specifically how gaps in housing, transportation and health planning in the city produce marginalization among Black youth and their families. Further research could explore how territorial stigma is produced to not only influence encounters with the police but shape Black youth’s coping strategies during and after said encounters. Moreover, understanding the multiple meanings of space for Black youth and their families is central to further investigating the ambiguity of community for those who go through the criminal justice system. This is crucial in identifying stakeholders in the rehabilitation process and advocating for more spatially sensitive approaches to mitigating the impact of anti-Black racism on Black families across Canada.

Ultimately, this exploratory study contributes vital findings to the research on Black youth and criminalization in the Canadian context. The weathering effect of encounters with the criminal justice system, hypervigilance and sense of insecurity and the limits of community are identified as key themes from the experiences of Black youth and their families with direct and indirect experiences with the criminal justice and child welfare systems. More research employing a critical, intersectional approach, as we have proposed, will further nuance our understanding of how policing in Canadian cities impacts the mental health of racialized communities, and inform the design of best practices for culturally sensitive service provision.

## Data Availability

The original contributions presented in the study are included in the article/supplementary material, further inquiries can be directed to the corresponding author/s.
